# Polarization-sensitive optical responses from natural layered hydrated sodium sulfosalt gerstleyite

**DOI:** 10.1038/s41598-022-08235-8

**Published:** 2022-03-10

**Authors:** Ravi P. N. Tripathi, Xiaodong Yang, Jie Gao

**Affiliations:** 1 Department of Mechanical and Aerospace Engineering, Missouri University of Science and Technology, Rolla, MO 65409 USA; 2 Department of Mechanical Engineering, Stony Brook University, Stony Brook, NY 11794 USA

**Keywords:** Integrated optics, Two-dimensional materials

## Abstract

Multi-element layered materials have gained substantial attention in the context of achieving the customized light-matter interactions at subwavelength scale via stoichiometric engineering, which is crucial for the realization of miniaturized polarization-sensitive optoelectronic and nanophotonic devices. Herein, naturally occurring hydrated sodium sulfosalt gerstleyite is introduced as one new multi-element van der Waals (vdW) layered material. The mechanically exfoliated thin gerstleyite flakes are demonstrated to exhibit polarization-sensitive anisotropic linear and nonlinear optical responses including angle-resolved Raman scattering, anomalous wavelength-dependent linear dichroism transition, birefringence effect, and polarization-dependent third-harmonic generation (THG). Furthermore, the third-order nonlinear susceptibility of gerstleyite crystal is estimated by the probed flake thickness-dependent THG response. We envisage that our findings in the context of polarization-sensitive light-matter interactions in the exfoliated hydrated sulfosalt layers will be a valuable addition to the vdW layered material family and will have many implications in compact waveplates, on-chip photodetectors, optical sensors and switches, integrated photonic circuits, and nonlinear signal processing applications.

## Introduction

Ultrathin vdW layered materials with their unique properties such as weak interplanar bonding, atomic-layer thickness, dangling-bond-free surface, interlayer coupling, and tailored electronic and optical responses have emerged as a promising platform for driving light-matter interactions beyond subwavelength scale^1,2^. Several important applications including sensors^3^, memory devices^4^, photovoltaics^5^, atomically-thin holograms^6^, electrically and optically modulated high harmonic generation^7–9^ based on vdW layered materials have been demonstrated to substantiate their prospects till date. Noticeably, these applications are primarily realized on traditional mono- and binary-element vdW layered materials such as graphene, black phosphorous, GeAs, ReS_2_ and transition metal dichalcogenides, therefore the material-associated inherent limitations such as elementary crystal structures, simple chemical compositions, material-dependent electronic and optical responses make the applications relatively monotonous and further confine their adaptability^10–13^. To address these limitations of mono- and binary-element materials, multi-element vdW layered materials^14,15^ facilitate an alternative and versatile testbed for realizing innovative electronic, photonic and optoelectronic applications^10–12^, according to their complex crystal structures, stoichiometric variations, and exceptional physical and chemical properties such as high charge carrier mobility^10,16^, favorable band structure^17^, unique phonon vibrations^11,17^, linear dichroism transition^18,19^, and robust air stability. In recent years, complex vdW layered materials have established their prospects via their engagement in many important applications. For example, franckeite (Pb_5_Sn_2_FeSb_2_S_14_) and cylindrite (Pb_3_Sn_4_FeSb_2_S_14_) nanosheets have been explored in the field of electrocatalyst^20^ and near-infrared photodetection^21,22^, whereas Ta_2_PdS_6_^23^, Ta_2_NiS_5_^24^, and Bi_2_O_2_Se^25^ nanosheets have been investigated for photodetection, broadband optical pulse generation, spin–orbit coupling and magneto-transportation.

Recently, complex multi-element vdW layered materials have attracted vast attention in the context of anisotropic linear and nonlinear optical responses^18,26–30^. Since the anisotropic optical responses are prominently material specific, it is obligatory to explore more new types of multi-element vdW materials for thriving their unique physical properties and increased adaptability and versatility in more applications. Motivated with this, herein we introduce alkaline sulfosalt gerstleyite as an anisotropic optical material to further expand the existing two-dimensional (2D) vdW material library. Sulfosalts represent a family of naturally occurring complex mixed-metal sulfide^31,32^. Gerstleyite is a quite rare hydrated sodium antimony arsenic sulfosalt, with the idealized chemical formula of Na_2_(Sb,As)_8_S_13_⋅2H_2_O, which was first described in 1956 from Baker mine in the Kramer borate district, California and named in honor of James Mack Gerstley, President of the Pacific Coast Borax Company^33^. Gerstleyite is formed by highly alkaline low-temperature hydrothermal fluids, and it occurs as cinnabar-red fibrous and platy spherules embedded in clay with borates. It is translucent and has a brittle tenacity with a Mohs scale hardness of 2.5. In contrast to conventional vdW materials, gerstleyite are recognized as a rarely available unique alkaline sulfosalt, hydrated with water molecules. Such hydrated layered materials with interlayer cations (such as Na^+^ and K^+^) and rapid ion transport kinetics have recently been identified as a suitable candidate for energy storage, renewable energy source and other electrochemical applications^15,34^. Despite having such a vibrant preface, little attention has been devoted on exploring the optical properties of these hydrated layered materials. Moreover, the effective understanding of their optical responses in the context of chemical composition, crystal structure, interlayer coupling, structural characteristics and layer thickness will certainly pave a way of their proficient engagement in future nanoscale device applications.

With this hindsight, polarization-sensitive anisotropic linear and nonlinear light-matter interactions in mechanically exfoliated gerstleyite thin flakes are systematically investigated in this work. It is demonstrated that naturally occurring hydrated alkaline sulfosalt bulk crystal can be mechanically exfoliated to ultrathin flakes of different thicknesses. The prepared gerstleyite flakes are characterized using atomic force microscopy (AFM), high-resolution transmission electron microscopy (HRTEM) and energy dispersive X-ray spectroscopy (EDXS) techniques to determine the flake thickness, surface smoothness, crystal structure and chemical composition. Next, we probe the anisotropic vibrational responses of gerstleyite flakes induced by the reduced in-plane crystal symmetry by using angle-resolved polarized Raman spectroscopy. Further, we explore the effect of the reduced crystal symmetry on linear optical response using polarization-resolved optical absorption spectroscopy. Interestingly, wavelength-dependent linear dichroism transition phenomena and strong birefringence effects are observed in gerstleyite flakes, which are relevant to future applications in compact optical waveplates, liquid crystal displays, photodetectors, and optical sensors. Finally, the polarization-resolved anisotropic THG is probed in gerstleyite flakes and the third-order nonlinear susceptibility is estimated by measuring the flake thickness-dependent THG emission. We envisage that these results will be valuable in harnessing the hydrated alkaline sulfosalt vdW materials in advancing on-chip polarization-sensitive photodetectors, optical sensors and switches, frequency modulators, nonlinear signal processing, and energy storage applications.

## Results

### Crystal structure and chemical composition of gerstleyite

**Figure 1:**
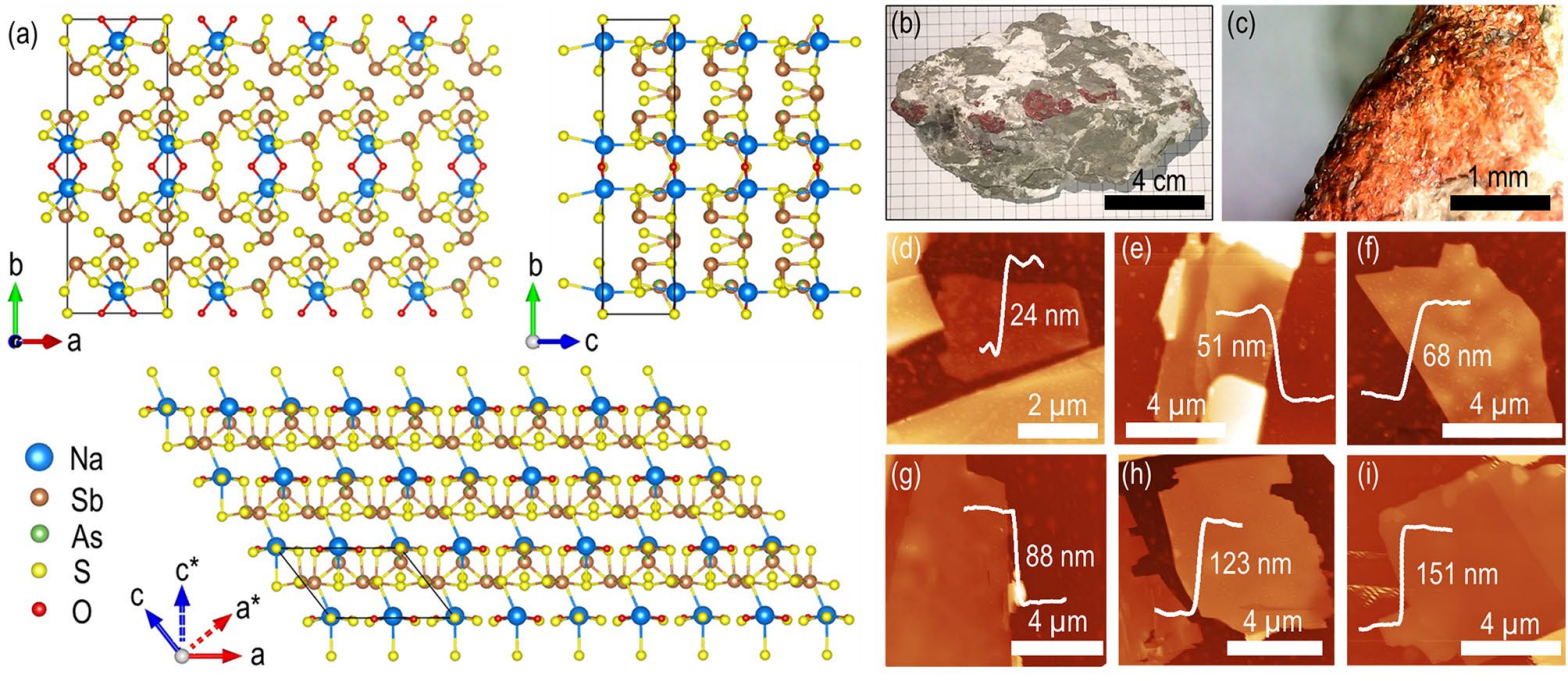
Gerstleyite crystal structure and flake exfoliation. (**a**) Projection of the gerstleyite crystal structure viewed along the *c*-axis, *a*-axis, and *b*-axis. (**b**) Picture of a natural bulk gerstleyite mineral rock. (**c**) Magnified view of one gerstleyite crystal area. (**d**–**i**) Captured AFM images of several mechanically exfoliated gerstleyite flakes, where the AFM line profiles signify the flake thickness and surface smoothness.

Gerstleyite belongs to the monoclinic crystal with space group *Cm* and the unit cell dimensions of *a* = 9.912 Å, *b* = 23.052 Å, *c* = 7.098 Å, *α* = *γ* = 90° and *β* = 127.86°^35^. As presented in Fig. 1a, in the crystal structure of gerstleyite, all Sb atoms are essentially three-coordinated by S atoms with short Sb–S bonds, forming trigonal SbS_3_ pyramids. The trigonal SbS_3_ pyramids share corners to form complex Sb–S double chains running in [100] along the *a*-axis. The Na–S bonds cross-link the Sb–S double chains to form a slab structure parallel to (010) along the *b*-axis. The water molecules are bound only to two Na atoms. The slab structures are separated by long Sb–S distances across the zigzag interspace filled with lone electron pairs of Sb. The zigzag interspace is oriented along the a*-axis which has an angle of around 38° with respect to the *a*-axis. The crystal structure of gerstleyite is homeotypic with those of ambrinoite (K,NH_4_)_2_(As,Sb)_8_S_13_·H_2_O^36^ and gillulyite Tl_2_(As,Sb)_8_S_13_^37^, all belonging to the hutchinsonite merotypic series. The chains of corner-sharing MS_3_ pyramids of gerstleyite are similar to those of ambrinoite and gillulyite. The difference is that in gerstleyite the two single chains within the double chain have a mirror plane, whereas such mirror symmetry is not present in ambrinoite and gillulyite. The weak interactions between the slab structures of gerstleyite will result in a low interlayer cohesive energy, which indicates the feasibility of mechanical exfoliation of the bulk mineral. Here the bulk natural gerstleyite mineral (from Western Mine, Boron, Kern County, California, USA) is mechanically exfoliated with Nitto tape (SPV 224) to obtain thin flakes with different thicknesses on glass substrate. Figure 1b shows a picture of a gerstleyite mineral rock, where an aggregate of deep-red granular and platy crystals of gerstleyite is embedded in the gray-green clay matrix with white sodium borate. The corresponding magnified view of one crystal area is displayed in Fig. 1c. Figure 1d–i show the AFM images of several mechanically exfoliated gerstleyite flakes with thicknesses of 24, 51, 68, 88, 123 and 151 nm, underlining the high aspect ratios and surface smoothness of the exfoliated crystals.

**Figure 2:**
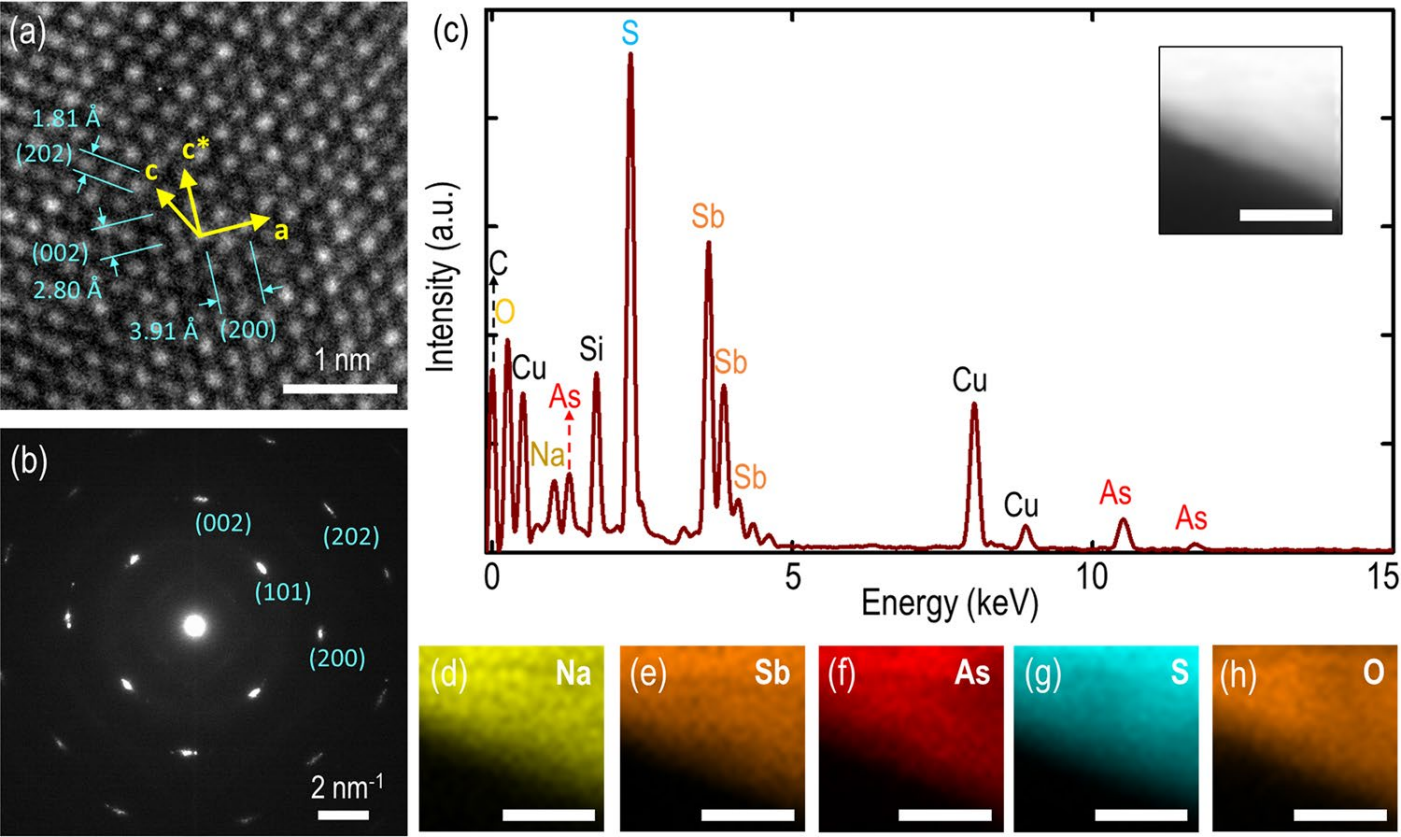
Atomic arrangement and chemical composition analysis. (**a**) Captured HRTEM image of an ultrathin gerstleyite crystal. (**b**) Corresponding SAED pattern emphasizing the crystalline nature of probed crystal. (**c**) Recorded average EDXS spectrum underlining the presence of prime elements (Na, Sb, As, S and O) in the examined crystal. The inset shows the dark-field TEM image of a crystal section used for capturing TEM-EDXS elemental maps. (**d**–**h**) TEM-EDXS elemental maps of major elements, emphasizing the homogenous distributions of these elements throughout the crystal. The scale bar indicates 50 nm.

The gerstleyite crystal is further investigated to analyze the crystallinity nature, atomic arrangement and chemical composition using HRTEM and EDXS. Figure 2a shows a captured HRTEM image of gerstleyite crystal with the determined lattice spacings of ~ 3.91 Å and ~ 2.80 Å, which are consistent with the [200] and [002] sets of planes for the monoclinic crystal. The intersection angle between the *a*-axis and *c*-axis is ~ 120°. The captured selected area electron diffraction (SAED) pattern is shown in Fig. 2b, which further validates the crystalline nature of the exfoliated gerstleyite flakes where the spot patterns from the surface normal to the [010] crystal zone axis are displayed. The chemical composition of gerstleyite crystal is further quantified. Figure 2c shows the recorded average EDXS spectrum, emphasizing the presence of all the prime elements of sodium (Na), antimony (Sb), arsenic (As), sulfur (S), and oxygen (O) in the probed specimen. The absence of hydrogen (H) element is attributed to its lightweight and low efficiency of EDXS process. Besides, there are several other peaks such as copper (Cu), carbon (C), and silicon (Si) can also be noticed in the acquired EDXS spectrum. The observed Cu peaks in the EDXS spectrum are contributed from the copper TEM grid, whereas the presence of C and Si impurities is expected in naturally occurring vdW materials due to their geological origins^20,22^. To further validate the chemical composition, TEM-EDXS elemental maps are performed on the probed specimen. The inset of Fig. 2c shows the dark-field TEM image of one scanned crystal region and the corresponding TEM-EDXS elemental maps are illustrated in Fig. 2d–h, emphasizing the homogenous distributions of prime components in Na, Sb, As, S and O. In addition, the recorded EDXS elemental maps of copper and carbon validate our claim that these signals are contributed from the TEM grid and the underlying carbon film support. The elemental composition is further summarized in Table 1, which is used to analyze the compositional stoichiometry of gerstleyite crystal, giving an empirical formula of Na_3.35_Sb_7.05_As_2.03_S_13.00_. Here, it can also be observed that the empirical formula determined from EDXS is not completely matched with the generic gerstleyite chemical formula of Na_2_(Sb,As)_8_S_13_. The small mismatch in the empirical formula determined from the EDXS spectrum and the generic chemical formula is attributed to the effects from several factors including the overlap in X-ray emission peaks, the low detection efficiency, and the nature of probed specimen.

**Table 1:** EDXS quantification of bulk gerstleyite.

Elements	Concentration (at%)
Na	13.24 ± 3.51
Sb	27.69 ± 1.53
As	7.98 ± 1.68
S	51.09 ± 2.08

### Anisotropic Raman vibrational modes in gerstleyite and crystal axis determination

**Figure 3:**
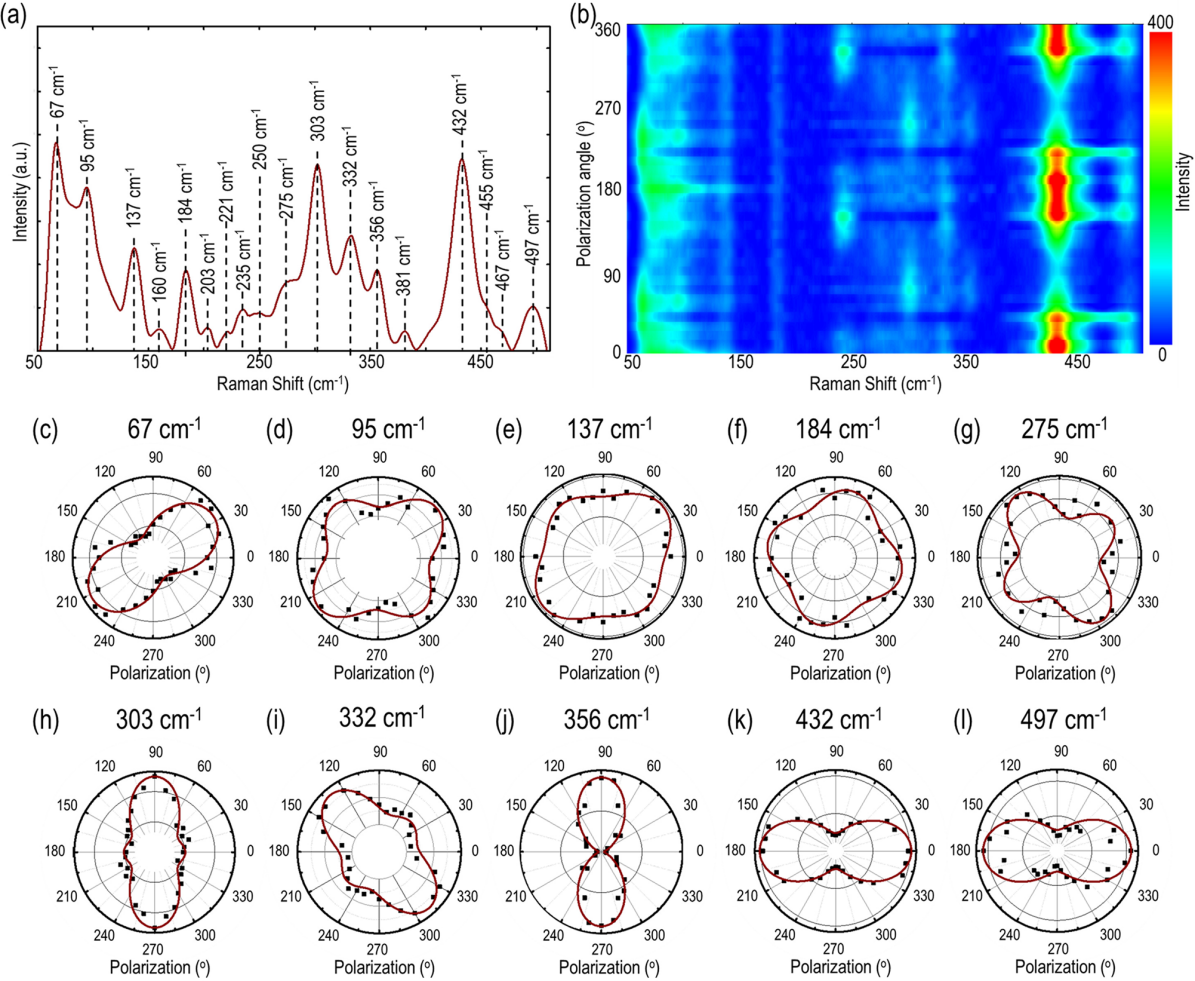
Evolution of angle-resolved Raman modes in gerstleyite crystal. (**a**) Recorded Raman spectra of the 88 nm-thick gerstleyite flake. The identified Raman modes are indicated with black dashed lines. (**b**) Color map of the recorded angle-resolved Raman spectra in parallel polarization configuration. (**c**–**l**) Polar plots of the Raman intensity variations of parallel polarization components for Raman modes at 67, 95, 137, 184, 275, 303, 332, 356, 432 and 497 cm^−1^. Black squares indicate the measured data points, while brown solid lines show the corresponding theoretical fits.

The reduced in-plane crystal symmetry of gerstleyite emphasizes on the significant dependency of Raman vibrational mode intensity on incident polarization angle. To understand the evolution of vibrational modes, angle-resolved polarized Raman spectroscopy is performed on the 88 nm-thick flake excited with a 632.8 nm He–Ne laser beam in parallel polarization configuration, where the direction of linear analyzer in the collection path is parallel to the incident linear polarization. Figure 3a shows the recorded Raman spectrum of gerstleyite crystal in parallel polarization configuration, presenting multiple distinct Raman peaks in the 50–500 cm^−1^ range located at 67, 95, 137, 160, 184, 203, 221, 235, 250, 275, 303, 332, 356, 381, 432, 455, 467, and 497 cm^−1^. The Raman modes of gerstleyite can be assigned according to the Raman spectra of its component binary sulfides, including stibnite Sb_2_S_3_^38,39^, orpiment As_2_S_3_^40–42^, realgar As_4_S_4_^43,44^, and sodium polysulfides Na_2_S_2_, Na_2_S_3_, Na_2_S_4_ and Na_2_S_5_^45,46^. The 67 cm^−1^ peak is assigned as the Raman modes of As_2_S_3_ (69 cm^−1^), Sb_2_S_3_ (62 cm^−1^) and Na_2_S_2_ (64 cm^−1^). The peak at 95 cm^−1^ is attributed to the A_g_ mode of Sb_2_S_3_ (101 cm^−1^) and the Raman mode of Na_2_S_4_ (97 cm^−1^). The 137 cm^−1^ peak corresponds to a combination of the A_g_ mode of As_2_S_3_ (136 cm^−1^), the E_1g_ mode of Na_2_S_2_ (134 cm^−1^) and the torsion mode of Na_2_S_5_ (135 cm^−1^). The 160 cm^−1^ peak represents both the A_g_ mode of As_2_S_3_ (154 cm^−1^) and the A_g_ mode of Sb_2_S_3_ (156 cm^−1^). The 184 cm^−1^ peak is attributed to a combination of the Raman modes of As_4_S_4_ (183 cm^−1^) and Sb_2_S_3_ (191 cm^−1^). The peak at 203 cm^−1^ is assigned as both the A_g_ mode of As_2_S_3_ (203 cm^−1^) and the symmetric bending mode of Na_2_S_4_ (206 cm^−1^). The peak at 221 cm^−1^ corresponds to both the Raman mode of As_4_S_4_ (221 cm^−1^) and the bending mode of Na_2_S_5_ (214 cm^−1^). The 235 cm^−1^ peak belongs to the Raman mode of As_4_S_4_ (235 cm^−1^), the B_1g_/B_3g_ mode of Sb_2_S_3_ (238 cm^−1^), the Raman mode of Na_2_S_3_ (238 cm^−1^) and the asymmetric bending mode of Na_2_S_4_ (239 cm^−1^). The 250 cm^−1^ peak is attributed to the bending mode of Na_2_S_5_ (266 cm^−1^). The peak of 275 cm^−1^ is assigned as the A_g_ mode of Sb_2_S_3_ (283 cm^−1^). The peak of 303 cm^−1^ corresponds to the A_g_ mode of As_2_S_3_ (311 cm^−1^) and the A_g_ mode of Sb_2_S_3_ (312 cm^−1^). The 332 cm^−1^ peak is attributed to the Raman mode of As_4_S_4_ (327 cm^−1^). The peak at 356 cm^−1^ represents the combined Raman modes of As_2_S_3_ (355, 359 cm^−1^) and As_4_S_4_ (354 cm^−1^). The peak of 381 cm^−1^ is assigned as the Raman modes of As_2_S_3_ (382 cm^−1^) and As_4_S_4_ (375 cm^−1^). The 432 cm^−1^ peak corresponds to the stretching mode of Na_2_S_5_ (429 cm^−1^) and the A_1g_ mode due to isotopic sulfur species of Na_2_S_2_ (442 cm^−1^). The 455 cm^−1^ peak belongs to a combination of the A_1g_ mode of Na_2_S_2_ (451 cm^−1^), the stretching mode of Na_2_S_3_ (458 cm^−1^) and the symmetric stretching mode of Na_2_S_4_ (445 cm^−1^). The 467 cm^−1^ peak represents the asymmetric stretching mode of Na_2_S_4_ (468 cm^−1^). The peak at 497 cm^−1^ is assigned as the symmetric stretching mode of Na_2_S_4_ (482 cm^−1^) and the stretching mode of Na_2_S_5_ (488 cm^−1^). Next, the incident linear polarization is continuously varied to obtain further insight about the anisotropic nature of the observed Raman modes. Figure 3b plots the color map of the recorded angle-resolved Raman spectra in parallel polarization configuration as a function of the incident linear polarization angle for the 88 nm-thick flake. It is observed that the intensities of Raman modes show periodic variations as a function of the incident polarization angle.

To get further insight regarding the evolution of angle-resolved Raman scattering intensity, the theoretical framework is utilized to fit the measured Raman modes. In general, the Raman intensity can be understood in the context of the Raman tensor (*R*) related to the crystal symmetry together with the unit polarization vectors of the incident and scattered beam ${e}_{i}$ and ${e}_{s}$, as $I \propto {\left|{e}_{i}\cdot R\cdot {e}_{s} \right|}^{2}$. In our case, gerstleyite crystal belongs to the monoclinic crystal family, which can support both A_g_ and B_g_ modes. The corresponding Raman tensor for different A_g_ and B_g_ modes can be written as^18^

1 $$R \left({A}_{g}\right)= \left[\begin{array}{ccc}a& 0& d\\ 0& b& 0\\ d& 0& c\end{array}\right],$$

2 $$R \left({B}_{g}\right)= \left[\begin{array}{ccc}0& e& 0\\ e& 0& f\\ 0& f& 0\end{array}\right],$$

where *a*, *b*, *c*, *d*, *e*, and *f* are the amplitudes of Raman tensor elements with the associated complex phase factors. Taken into consideration of ${e}_{i}=(\cos\theta , \sin\theta , 0)$ and ${e}_{s}=(\cos\theta , \sin\theta , 0)$ in parallel polarization configuration, the resultant Raman intensity for A_g_ and B_g_ modes can be expressed as

3 $${I}_{{A}_{g}}^{//}\propto {\left(\left|a\right|{\sin}^{2}\theta +\left|b\right|{\cos}^{2}\theta \right)}^{2}+{\left(\left|b\right|{\cos}^{2}\theta \right)}^{2},$$

4 $${I}_{{B}_{g}}^{//} \propto {\left|e\right|}^{2} {\sin}^{2}2\theta .$$

Figure 3c–l show the polar plots of the Raman modes at 67, 95, 137, 184, 275, 303, 332, 356, 432 and 497 cm^−1^. The measured values indicated with black squares match well with the corresponding theoretical fittings in brown solid lines calculated from Eqs. (3) and (4). Among these observed Raman modes, the 184 cm^−1^ one is acknowledged as a B_g_ mode, whereas all the others are identified as A_g_ modes. Noticeably, the complex crystal structure of gerstleyite plays a crucial role in determining the Raman mode axis. As a result, the observed vibrational modes are aligned along two different orientations according to the crystal symmetrical axis at 0° and 90° (*a-*axis and c*-axis), or 38° and 128° (a*-axis and *c*-axis), as illustrated in Fig. 1a. The A_g_ modes at 67, 95, 137, 275 and 332 cm^−1^ show anisotropic two-lobe patterns with a period of 180° , where the Raman intensity primary maxima are located at either ~ 38° and 218° in the zigzag interspace direction along the a*-axis (Fig. 3c–e), or ~ 128° and 308° in its perpendicular direction along the *c*-axis (Fig. 3g,i), which are mainly due to the contributions from the Sb–S double chains separated by the zigzag interspace. Whereas other A_g_ modes around 303, 356, 432 and 497 cm^−1^ exhibit anisotropic two-lobes patterns with the primary maxima aligned in either ~ 90° and 270° along the c*-axis (Fig. 3h,j), or ~ 0° and 180° along the *a-*axis (Fig. 3k,l). It is noted that the anisotropic Raman modes at 432 and 497 cm^−1^ oriented along the *a*-axis are attributed to the Na–S bonds in the middle of the slab structure. In contrast, the B_g_ mode at 184 cm^−1^ exhibits the characteristic four-lobe pattern with a period of 90° and the Raman intensity maxima located ~ 75°, 165°, 255° and 345° (Fig. 3f). The measured values are shown with black squares, while the corresponding fits are indicated with brown solid lines. Based on this, it is inferred that the Raman vibrational modes in gerstleyite crystal are highly anisotropic in nature and the orientations of vibrational modes are significantly affected by its specific crystal structural symmetry. It is noted that although both gerstleyite and gillulyite belong to the monoclinic crystal system and the crystal structure of gerstleyite is homeotypic with that of gillulyite, the Raman spectra are different from each other. Both A_g_ and B_g_ modes are observed in gerstleyite, while only A_g_ modes are found in gillulyite within the 50–420 cm^−1^ frequency range^30^. One reason for that is the distinguishable crystal structures of gerstleyite and gillulyite, where the double chain in gerstleyite has a mirror symmetry but it is not present in gillulyite. Beside the crystal symmetry, the evolution of different types of Raman modes also depends on several other important factors such as bond strength, dipole moment, and electron mobility in bonds, which are completely material specific. Here, not only there is difference between Na contained in gerstleyite and Tl in gillulyite, but it is also important to note that Sb plays a dominant role in determining the crystal structure of gerstleyite, whereas As shows its dominance in gillulyite. This underlines that the intrinsic nature of both the crystals are different to introduce the dissimilar bond polarizability in the evolution of Raman modes and hence the unidentical Raman scattering responses under certain excitation energy. In addition, these observed anisotropic Raman modes also suggest the presence of strong linear dichroism in gerstleyite crystal.

### Linear dichroism transition in gerstleyite crystal

**Figure 4:**
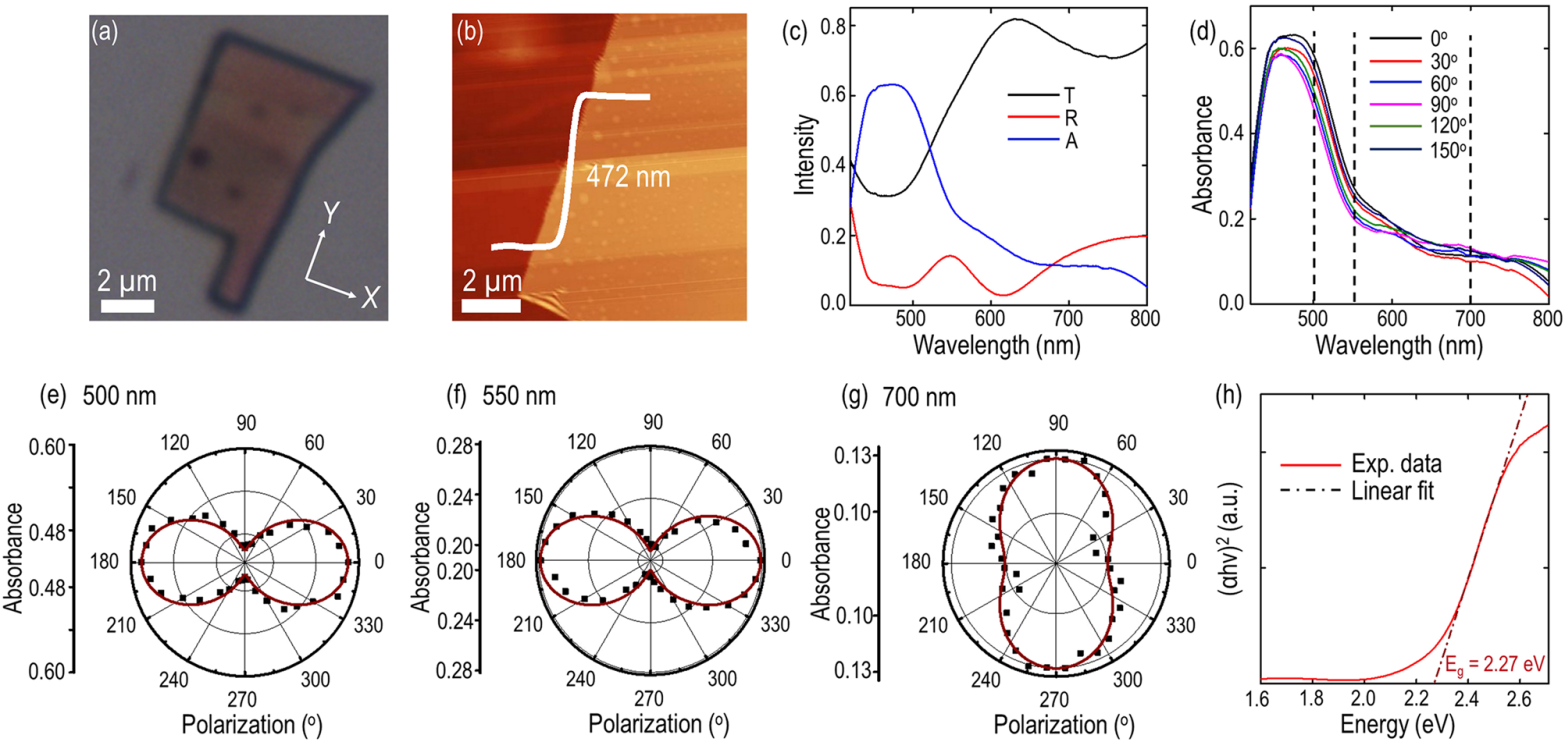
Anisotropic linear optical response and linear dichroism transition. (**a**) Optical transmission microscope image of the investigated gerstleyite flake. (**b**) Captured AFM image of the flake. (**c**) Recorded reflectance (*R*), transmittance (*T*), and absorbance (*A*) spectra. (**d**) Measured polarization-resolved absorbance spectra under incident linear polarization angle from 0° to 180°. (**e**–**g**) Evolution of absorbance as a function of the incident polarization angle at three different wavelengths at 500, 550 and 700 nm indicated with black dashed lines in (**d**). The measured data points are denoted with black squares, whereas the theoretical fits are signified with brown solid lines. (**h**) Measured Tauc plot and direct optical band gap extraction.

The anisotropic linear optical response in gerstleyite flake is recorded using polarization-resolved optical absorption spectroscopy. Initially, the optical absorption spectrum from bare glass substrate is characterized to elucidate the substrate effect on the measurement. The optical absorption spectra from the 472 nm-thick flake are probed in the visible range from 420 to 800 nm. Figure 4a,b show the optical transmission microscope image and the corresponding AFM image of the flake. The measured reflectance (*R*), transmittance (*T*) and absorbance (*A* = 1 − *R − T*) spectra are plotted in Fig. 4c. It is observed that the transmittance is much higher than the reflectance. Both the transmittance and reflectance show sharp decline before the wavelength around 450 nm and then show gradual increase up to 510 nm. The opposite oscillations in the transmittance and reflectance spectra around the wavelength of 620 nm is attributed to the thin film interference of light within the flake. However, these oscillations are almost canceled out in the absorbance spectrum. It can be observed that the initial decline in transmittance and reflectance leads to the maximum absorbance before 510 nm. Afterwards the absorbance rapidly declines up to 570 nm followed by a gradual decrease continuing till 800 nm. To obtain further insight about the anisotropic feature of gerstleyite crystal, the polarization-resolved absorbance spectra are systematically recorded for the incident linear polarization angle from 0° to 180° as shown in Fig. 4d, where the linear polarization angle is measured relative to the crystal’s *a*-axis (*x*-axis as depicted in Fig. 4a). The absorbance spectra show the significant dependence on the incident linear polarization. Interestingly, all the absorbance spectra from 0° to 180° converge around 650 nm and a clear crossover around this wavelength can be observed. Further, the polar plots of absorbance at three different wavelengths of 500, 550 and 700 nm are shown in Fig. 4e–g. It is found that the absorbance maxima at 500 and 550 nm in Fig. 4e,f are aligned in 0° and 180° along the *a-*axis (*x*-axis), which are consistent with the observed Raman A_g_ modes at 432 and 497 cm^−1^ (Fig. 3k,l). Whereas the absorbance maxima at 700 nm in Fig. 4g is observed to be 90° rotated along the c*-axis (*y*-axis), which follows the orientations of the Raman A_g_ modes at 303 and 356 cm^−1^ (Fig. 3h,j). Such observation infers that the optical axis of gerstleyite crystal is along the *a*-axis and c*-axis. The measured absorbance values marked in black squares show two-lobe patterns and can be theoretically fitted as brown solid lines using a sinusoidal function expressed as $A \left(\theta \right)= {A}_{x} {\cos}^{2}\left(\theta \right)+ {A}_{y} {\sin}^{2}\left(\theta \right)$, where ${\alpha }_{x}$ and ${\alpha }_{y}$ are the absorbance magnitudes along *x*-axis and *y*-axis and $\theta$ is the linear polarization angle. The obtained absorbance anisotropy ratios *A*_*x*_*/A*_*y*_ at 500, 550 and 700 nm are retrieved as 1.4, 1.3, and 0.77 respectively, signifying the presence of strong linear dichroism in the crystal. Importantly, the linear dichroism in gerstleyite crystal also validates the existence of unbalanced energy band dispersion along different crystalline axes, which results in anisotropic photon energy absorption along the probed crystal directions of *a*-axis and c*-axis. Intriguingly, we also observe the absorbance anisotropy ratio at 700 nm of less than 1, which endorses that the probed crystal undergoes the reversibility of linear dichroism polarity around 650 nm, resulting in linear dichroism transition phenomenon. Such linear dichroism transition can be understood in the context of singularities in the joint density of states induced strong localization of the absorption peaks along two principal crystal directions in the energy band at the specific photon energies^19,28^. Such interesting observations have also been reported in several other 2D crystals such as palladium diselenide PdSe_2_, BaTiS_3_ and getchellite^18,19,28^. Nevertheless, further studies correlating the gerstleyite crystal band structure and the associated optical transitions are essential to obtain insight about this interesting phenomenon.

In addition, the stoichiometric variation in complex vdW 2D materials can substantially modulate the optical band gap and band structure. The optical band gap (${E}_{g}$) of gerstleyite crystal is estimated by the Tauc plot using the relation ${\left(\alpha h\nu \right)}^{n}=A(h\nu -{E}_{g})$, where $\alpha$ signifies the absorption coefficient corresponding to the photon energy $h\nu$ ($h$ is the Plank’s constant and $\nu$ is the incident photon frequency), ${E}_{g}$ denotes optical band gap energy and $A$ is a constant. The value of $n=2$, and $1/2$ indicate an allowed direct transition and indirect transition, while $n=2/3$ denotes the forbidden transition. The value of $\alpha$ can be calculated with the measured transmittance and reflectance from the investigated flake of thickness $d$ using the expression^47^

5 $$\alpha =\frac{1}{d}ln\left\{\frac{2T}{{\left[{T}^{2}-{\left(1-R\right)}^{2}\right]+\left\{{\left[{T}^{2}-{\left(1-R\right)}^{2}\right]}^{2}+4{T}^{2}\right\}}^{1/2}}\right\}.$$

Figure 4h shows the plot of ${\left(\alpha h\nu \right)}^{2}$ as a function of the incident photon energy ($h\nu$). The obtained plot has a section of straight line indicating an allowed direct transition and hence the presence of direct band gap. Further, this section can be linearly fitted and extended to the photon energy axis as shown in the dash-dotted line in Fig. 4h. The intercept value at the photon energy axis of the fitted line provides an estimation of the optical band gap ~ 2.27 eV for the probed gerstleyite flake. It is noteworthy that the obtained band gap value (~ 2.27 eV) resides in between the band gaps of its constituent binary sulfide semiconductor materials of Na_2_S_5_ (~ 1.73 eV)^48^, Sb_2_S_3_ (~ 1.72 eV)^49^, As_2_S_3_ (~ 2.37 eV)^50^ and As_4_S_4_ (~ 2.40 eV)^51^, which emphasizes its consistency with the previously reported optical band gaps of multi-element sulfosalt minerals^52^.

### Birefringence in anisotropic gerstleyite crystal

**Figure 5:**
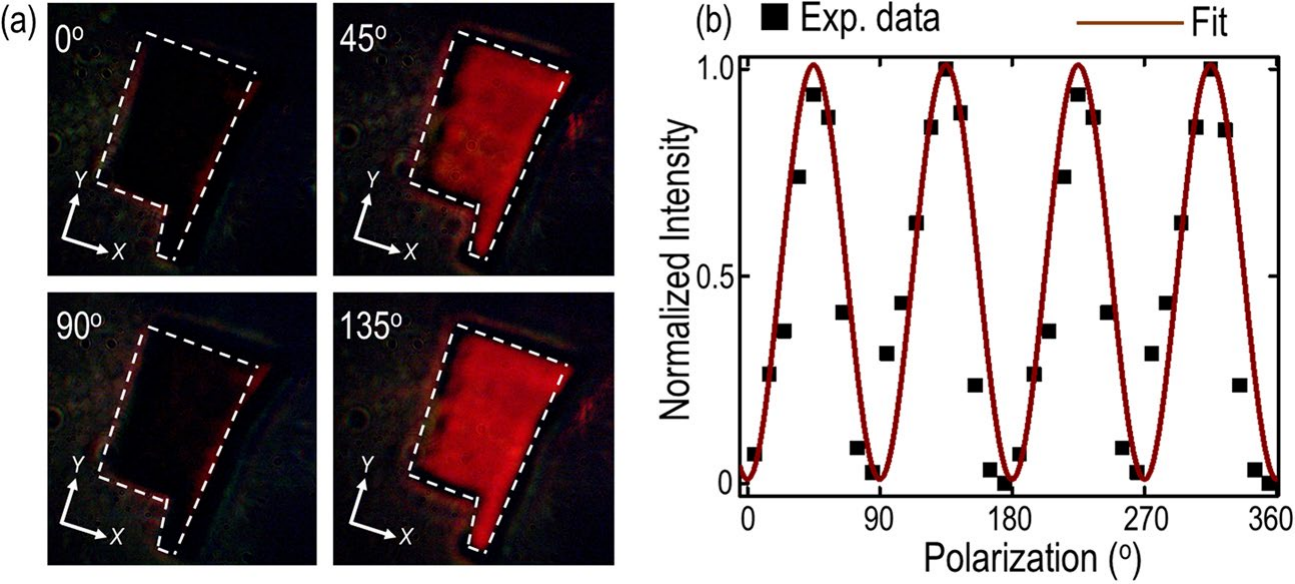
Birefringence effect in gerstleyite crystal. (**a**) Captured optical transmission microscope images of the 472 nm-thick gerstleyite flake under the cross-polarization illumination configuration at four different incident linear polarization angles of 0°, 45°, 90° and 135°. The investigated gerstleyite flake boundary is indicated with white dashed lines. The other experimental parameters such as incident beam intensity and image exposure time are kept same during the measurement. (**b**) Quantification of the transmitted light intensity as a function of the incident linear polarization angle under the cross-polarization illumination configuration. Black squares signify the experimentally extracted values, whereas brown solid curve shows the theoretical fitting.

The existence of strong linear dichroism further emphasizes the anisotropic refraction properties in gerstleyite crystals as well. Therefore, we further examine the birefringence in gerstleyite crystal using polarization-resolved optical microscopy. The linear polarization of incident white light is varied from 0° to 180° in a step of 10°, while positioning the directions of linear polarizer in the excitation path and linear analyzer in the collection path perpendicular to each other. Figure 5a shows the transmitted polarization-resolved optical microscope image of the 472 nm-thick gerstleyite flake at the incident polarization angle of 0°, 45°, 90°, and 135°. It can be observed that the brightness of the probed flake is highest when the crystal’s optical axis of *a*-axis and c*-axis which are labeled as *x*-axis and *y*-axis in Fig. 5a is aligned at the 45° and 135° incident polarization direction, compared to the case when the crystal’s optical axis is orientated at the 0° and 90° incident polarization direction. Such periodic variation in flake brightness is attributed to the birefringence effect of anisotropic gerstleyite crystal.

This observed birefringence effect can be ascribed in the context of the outcoupled polarization state of transmitted light^53,54^. When the incident linear polarization is aligned with the probed crystal’s optical axis (*a*-axis and c*-axis), the outcoupled polarization state of transmitted light remains unperturbed. As a result, the output polarization is still perpendicular to the direction of the engaged linear analyzer in the collection path so that the transmitted image appears dark. In contrast to this, when the alignment of incident linear polarization is away from the crystal’s optical axis, the transmitted light passing through the probed crystal experiences the phase retardance and then the outcoupled polarization state of transmitted light transforms into elliptically polarized. The intensity of such phase retardance induced elliptically polarized light will obtain the maximum value at the incident linear polarization angle of 45° and 135°. Therefore, the flake brightness exhibits periodic variation as a function of the incident linear polarization angle with the maxima at 45° and 135° and the minima at 0° and 90°. To further quantify and reaffirm this trend, the transmitted images are processed by normalizing the background intensity. Furthermore, the brightness contrast between flake and glass substrate is computed at different illumination light polarization. Figure 5b plots the transmitted light intensity as a function of the incident linear polarization angle under the cross-polarization illumination configuration. The measured values are shown with black squares, whereas the theoretical fitting is shown with brown solid line according to the equation $T= t \cdot {\sin}^{2}(2\theta )$, where $t$ denotes the transmittance along crystal’s axis. The measured flake brightness demonstrates a four-lobes pattern with the maxima at 45° and 135°, as well as the minima at 0° and 90°. In addition, the experimental data shows a good agreement with the theoretical fit which future validates the observed birefringence effect.

### Quantification of polarization extinction ratio in gerstleyite crystal

**Figure 6:**
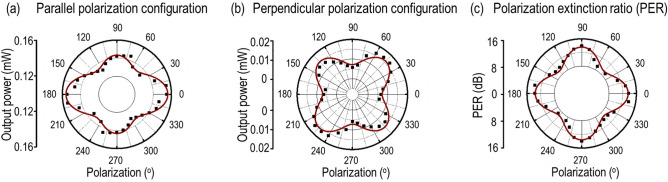
Quantification of polarization extinction ratio in gerstleyite flake. (**a,b**) Angle-resolved plots of transmission power variations in parallel and perpendicular polarization configurations through the 472 nm-thick gerstleyite flake. (**c**) Angular polar plot of *PER* values. The measured values are shown with black squares, while the fitted curves are shown with brown solid lines.

As one anisotropic vdW materials, gerstleyite has great potential for the application in polarization detection. The polarization extinction ratio (*PER*) is the key parameter to evaluate the polarization detection performance, which is defined as the ratio of the transmitted optical power in parallel (${P}^{//}$) and perpendicular (${P}^{\perp }$) polarization configurations for the incident linearly polarized beam propagating through a probed flake, and it can be expressed as $PER=10{\mathrm{ log}}_{10}({P}^{//}/{P}^{\perp })$ (in dB). The gerstleyite flake is illuminated with a 632.8 nm He–Ne laser source with a 40×, NA = 0.65 objective lens. The transmitted light is collected using a 100×, NA = 0.70 objective lens and directed towards a photodiode power sensor (S130C, Thorlabs). The incident linear polarization is controlled by introducing a linear polarizer and a half-wave plate in the excitation path. A linear polarizer analyzer is introduced in the collection path to further resolve the parallel and perpendicular polarization components of the transmitted light. Figure 6a,b show the plot of angle-resolved parallel and perpendicular components of the output power from the 472 nm-thick gerstleyite crystal. The measured values are indicated with black squares, while the theoretical fits are shown with brown solid lines. It can be observed that the transmitted signal in parallel polarization configuration exhibits a four-lobe pattern with the primary maxima along 0° and 180°, while the secondary maxima reside at 90° and 270°. Such anisotropic power values at 0° (~ 0.155 mW) and 90° (~ 0.142 mW) underline anisotropic absorption along two crystal axes of the gerstleyite flake, which is consistent with the linear dichroism results in Fig. 4. In contrast, the collected transmitted power in perpendicular polarization configuration validates our findings on the birefringence process in gerstleyite crystal. It is noticed that the transmitted power shows the characteristic four-lobe pattern with the maxima along 45°, 135°, 225° and 315° with almost equal power values. We further quantify the *PER* values of the probed crystal in Fig. 6c. It can be noted that the angular plot of *PER* has a typical four-lobe patten with the maxima residing along two crystal axes. The average *PER* value along the crystal axes (*x*- and *y*-axis) is obtained ~ 13.9 dB, while ~ 9.5 dB along 45° and 135° with respect to the crystal axes.

### Anisotropic nonlinear optical response and determination of third-order nonlinear susceptibility

**Figure 7:**
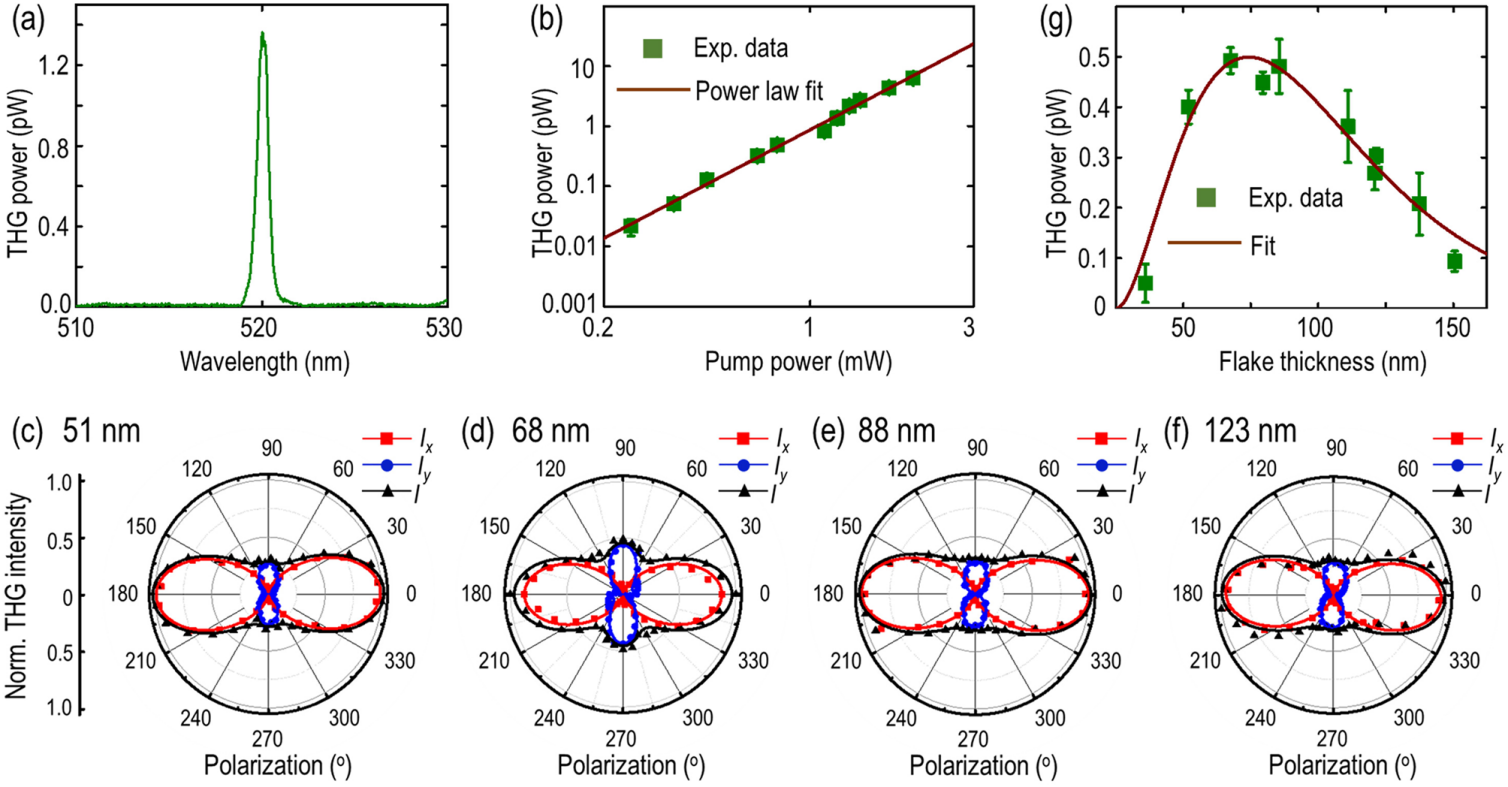
Anisotropic nonlinear optical response in gerstleyite crystal. (**a**) Collected THG emission spectrum with the peak wavelength at 520 nm from the 88 nm-thick gerstleyite flake. (**b**) Double log-scale plot of THG power as a function of the incident pump power. The linear fit with a slope of 3.00 (± 0.03) endorses the third-harmonic process. The measured values are indicated with green squares, while the power law fit is shown with brown solid line. (**c**–**f**) Angular dependence of THG power as a function of the incident linear polarization angle for four different gerstleyite flakes with thicknesses of 51, 68, 88 and 123 nm. The measured values along *x*-component (*I*_*x*_), *y*-component (*I*_*y*_) and total (*I*) are denoted with red squares, blue dots and black triangles, respectively. The corresponding theoretical fittings are shown with solid curves. (**g**) Evolution of THG emission power as a function of the gerstleyite flake thickness. The measured values are indicated with green squares and the corresponding theoretical fit is plotted as brown solid line.

The inherent reduced in-plane crystal symmetry also suggests the anisotropic nonlinear optical response to be present in such hydrated alkaline sulfosalt gerstleyite crystal. The THG emission process is investigated with a 1560 nm pulse laser with a beam waist of 1.5 µm. Figure 7a shows the recorded THG emission spectrum from the 88 nm-thick gerstleyite flake. It is observed that the intensity maxima peaks at 520 nm, which is exactly one-third of the incident pump beam wavelength. Afterwards, the THG emission process is reaffirmed in Fig. 7b with the cubic power law fit in the log-scale plot of the recorded THG emission power as a function of the input pump power. Next, the effect of crystal anisotropy on THG emission intensity is examined by varying the incident linear polarization of pump beam. As gerstleyite crystal belongs to monoclinic crystal structure, the contracted form of the corresponding third-order nonlinear susceptibility (${\chi }^{(3)}$) can be expressed as^55,56^

6 $${\chi }^{(3) }=\left[\begin{array}{ccc}{\chi }_{11}& 0& {\chi }_{13}\\ 0& {\chi }_{22}& 0\\ {\chi }_{31}& 0& {\chi }_{33}\end{array} \begin{array}{ccc}0& {\chi }_{15}& {\chi }_{16}\\ {\chi }_{24}& 0& 0\\ 0& {\chi }_{35}& {\chi }_{36}\end{array} \begin{array}{ccc}{\chi }_{17}& {\chi }_{18}& 0\\ 0& 0& {\chi }_{29}\\ {\chi }_{37}& {\chi }_{38}& 0\end{array} \begin{array}{c}0\\ {\chi }_{20}\\ 0\end{array} \right],$$

where the first term in subscript 1, 2 and 3 denotes *x*, *y* and *z* respectively and the second subscript refers to the combination of three components as

$$\begin{array}{ccc}xxx& yyy& zzz\\ 1& 2& 3\end{array} \begin{array}{ccc}yzz& yyz& xzz\\ 4& 5& 6\end{array} \begin{array}{ccc}xxz& xyy& xxy\\ 7& 8& 9\end{array} \begin{array}{c}xyz\\ 0\end{array}$$

Observing the experimental constraints, the linearly polarized electric field used to excite the gerstleyite crystal can be expressed as $\overrightarrow{E}=|E|(\cos\theta \widehat{x}+\sin\theta \widehat{y})$, where $\widehat{x}$, $\widehat{y}$ are the unit vectors along *x-*axis and *y-*axis, and $\theta$ is the linear polarization angle relative to the *a*-axis of the crystal. Since the excitation polarization always resides in the *x–y* plane, the ${\chi }^{(3)}$ elements containing *z*-components will no longer contribute to the measured THG emission. Thereby, only four non-zero ${\chi }^{(3)}$ tensor elements of ${\chi }_{11}$, ${\chi }_{18}$, ${\chi }_{22}$, ${\chi }_{29}$ will contribute and the resultant formula of THG intensity along *x-*axis and *y-*axis can then be expressed as

7 $${I}_{x}\propto {\left({\chi }_{11} {\cos}^{3}\theta + 3{\chi }_{18} \cos\theta\, {\sin}^{2}\theta \right)}^{2},$$

8 $${I}_{y}\propto {\left({\chi }_{22} {\sin}^{3}\theta + 3{\chi }_{29} \sin\theta\, {\cos}^{2}\theta \right)}^{2}.$$

Equations (7) and (8) are used to obtain the theoretical fit for the measured polarization-dependent THG response and further retrieve the relative magnitudes of the ${\chi }^{(3)}$ elements.

Figure 7c–f show the measured angular dependence of THG emission power with respect to the incident linear polarization angle for 51, 68, 88 and 123 nm-thick gerstleyite flakes, underlining the highly anisotropic two-lobe THG patterns for all the flakes. The primary maxima of THG emission are obtained with the incident linear polarization aligned along the crystal’s *a*-axis (*x*-axis), while the secondary maxima occur at the incident linear polarization along the c*-axis (*y*-axis). The red and blue data points denote the measured *x-* and *y-* components of THG power, whereas the black data points indicate the total THG power. The theoretical fits are shown with the solid lines in the corresponding color. Importantly, the observed good agreement between the measured THG intensity values and the theoretical fits further validate the anisotropic THG response of gerstleyite crystal and offer additional insights in its nonlinear optical properties, for instance the THG anisotropy ratio (${{|\chi }_{11}|}^{2}/{{|\chi }_{22}|}^{2}$) and the average relative magnitudes of ${\chi }^{(3)}$ elements. It is observed that the THG anisotropy ratio ${I}_{x}\left(\theta =0^\circ \right)/{I}_{y}(\theta =90^\circ )$ almost remains constant as ${{|\chi }_{11}|}^{2}/{{|\chi }_{22}|}^{2}=$ 3.37 for flakes with different thicknesses. Furthermore, the average relative magnitudes of ${\chi }^{(3)}$ elements are also retrieved as ${\chi }_{11}:{\chi }_{18}:{{\chi }_{22}:\chi }_{29}=1:0.195:0.545:0.083$, where the different magnitudes of ${\chi }^{(3)}$ elements endorse the prevailed structural anisotropy and its inherent nature of nonlinear optical properties in gerstleyite crystal.

In last, the third-order nonlinear susceptibility ${\chi }^{(3)}$ value of gerstleyite crystal is estimated by measuring the THG emission power as a function of the flake thickness. Owing to the presented insight^57^, the expression of the outcoupled THG power ${P}^{\left(3\omega \right)}$ as per the flake thickness can be obtained by solving the nonlinear Maxwell’s equations, and hence the third-order nonlinear susceptibility ${\chi }^{(3)}$ value can be expressed as^57^

9 $$\left|{\chi }^{\left(3\right)}\right|= {\left[\frac{16\sqrt{{n}_{3}^{2}+{k}_{3}^{2}}{n}_{1}^{3}{\epsilon }_{0}^{2}{c}^{4}{f}_{rep}^{2}{W}^{4}{\tau }^{2}{\left[\frac{\pi }{4\mathrm{ln}2}\right]}^{3}{P}^{\left(3\omega \right)}}{9{\omega }^{2}{d}^{2}{{P}^{\left(\omega \right)}}^{3}}\left(\frac{\left(\frac{4{\pi }^{2}{k}_{3}^{2}{d}^{2}}{{\lambda }_{3}^{2}} + {\Delta k}^{2}{d}^{2}\right)}{{e}^{-\frac{4\pi {k}_{3}d}{{\lambda }_{3}}}-2{\mathrm{\cos}(\Delta kd)e}^{-\frac{2\pi {k}_{3}d}{{\lambda }_{3}}}+1}\right){e}^{\frac{4\pi {k}_{3}d}{{\lambda }_{3}}}\right]}^{1/2}$$

where ${n}_{1}$, ${n}_{3}$ is the real part of the refractive index of gerstleyite crystal at the fundamental wavelength ${\lambda }_{1 }=1560\mathrm{ nm}$ and the THG wavelength ${\lambda }_{3 }=520$ nm, respectively. $\Delta k= \frac{6\pi }{{\lambda }_{1 }} ({n}_{1}- {n}_{3})$ is the phase mismatch between the fundamental beam and the forward propagating THG emission beam in the transmission optical setup arrangement. ${P}^{\left(\omega \right)}$ = 1.25 mW, *τ* = 90 fs, ${f}_{rep}$= 80 MHz, and *W* = 1.5 µm represent the experimental parameters of average pump power, laser pulse width, repetition rate, and spot size at the pump wavelength of 1560 nm, respectively. The average pump power is kept at 1.25 mW with 9.82 GW/cm^2^ peak irradiance. The THG emission power is measured with aligning the incident linear polarization along the *x*-axis. Figure 7g shows the measured THG emission power as a function of the probed gerstleyite crystal thickness. The measured values are shown with green squares with the respective error bars. It is observed that the THG emission power quickly increases up to 78 nm and afterwards shows an exponential decay of the THG signal for thicker gerstleyite flakes. This flake thickness-dependent THG signal variation can be explained with two competitive processes of optical gain and loss. For thin flakes, the THG emission power is proportional to the square of flake thickness, therefore the recorded THG signal shows continuous surge, as the optical absorption contribution in THG signal depletion is negligible. This trend can be observed from 24 to 78 nm flake thickness. However, for comparably thick flakes, the strong optical absorption at visible wavelength substantially attenuates the forward propagation of the emitted THG signal propagated through the flake. As a result, the THG signal exhibits an exponential decay as the flake thickness further increases greater than 78 nm. Nonetheless, such exponentially attenuated trend offers an estimation of the imaginary part of the refractive index *k*_*3*_ at ${\lambda }_{3}$= 520 nm for gerstleyite crystal by fitting the collected THG emission power with ${P}^{\left(3\omega \right)}\left(d\right)=A{d}^{2}\mathrm{exp}\left(-\frac{4\pi {k}_{3}d}{{\lambda }_{3}}\right)$, where *A* is a constant and *d* is the flake thickness. Figure 7g plots the exponentially fitted THG power with *k*_3_ = 0.85. From literature, the real part of the refractive index of gerstleyite crystal ${n}_{3}$ is around 2.01^58^. Thus, considering all the experimental parameters into account, the third-order nonlinear susceptibility magnitude of ${\chi }^{(3)}$ for gerstleyite crystal is estimated as 1.81 × 10^–20^ m^2^/V^2^, which has the same order of magnitude as the recently explored multi-element anisotropic nonlinear vdW layered materials for example franckeite (1.87 × 10^–19^ m^2^/V^2^)^26^, cylindrite (3.06 × 10^–19^ m^2^/V^2^)^27^, teallite (3.49 × 10^–19^ m^2^/V^2^)^29^, gillulyite (2.05 × 10^–20^ m^2^/V^2^)^30^, getchellite (2.89 × 10^–20^ m^2^/V^2^)^18^, and nagyágite (1.49 × 10^–20^ m^2^/V^2^)^59^.

## Discussion

In summary, we have mechanically exfoliated hydrated sulfosalt gerstleyite crystal flakes of different thicknesses. The crystal structure, chemical composition and the associated vibrational modes are comprehensively characterized. The observed vibrational modes are found to be highly anisotropic in nature and significantly influenced by the complex gerstleyite crystal structure. Furthermore, the linear optical responses of exfoliated gerstleyite crystals are investigated including linear dichroism transition, birefringence effect and optical band gap determination. Using polarization-resolved optical absorption spectroscopy, we demonstrate that linear optical responses of gerstleyite are highly anisotropic, which is ascribed to the prevailing low in-plane crystal symmetry. Moreover, in contrast to traditional layered materials, the anomalous wavelength-dependent linear dichroism polarity switching in gerstleyite crystal is observed. Next, the birefringence effect is demonstrated in gerstleyite flake. Such wavelength-dependent linear dichroism transition and birefringent effect observed in gerstleyite crystal can be utilized for prototyping future on-chip photonic devices of atomically-thin optical waveplates, liquid crystal displays, photodetectors and optical sensors. In addition, the direct optical band gap of gerstleyite crystal is identified as ~ 2.27 eV, endorsing the utilization of hydrated sulfosalt vdW layered materials in the context of photodiode, photovoltaic, and solar cell applications. Lastly, we have explored the anisotropic THG emission in gerstleyite crystal and extracted its third-order nonlinear susceptibility value of 1.81 × 10^–20^ m^2^/V^2^. The demonstrated strong anisotropic nonlinear optical response with a high THG anisotropy ratio of 3.37 in gerstleyite crystal endorses its engagement in future miniaturized photonic and optoelectronic applications such as integrated optical circuits, frequency conversion, wavelength‐division multiplexing, and nonlinear signal processing prototypes. In addition, we envision the integration of vdW layered materials and metasurfaces with their exceptional properties will provide a unique testbed for efficiently tailoring material responses by engineering light-matter interactions at subwavelength scale via controlled alterations in local photonic environment and local density of states using metasurfaces. Such hybrid architecture will also create a promising platform for further advancing many important applications such as holograms and electric and optical modulation^6,60–63^.

## Methods

### Sample preparation

The glass substrates (1 cm × 1 cm) are cleaned with acetone (99.9% Sigma-Aldrich), deionized water, and isopropyl alcohol (99.7% Sigma-Aldrich), followed by ultra-sonication and dried with N_2_ gas. This process is repeated several times to minimize the undesired residues and grease on the glass substrates. Further, these pretreated substrates are heat treated at 150–160 °C for 10 min to remove the solvent on the glass surface and kept it in low vacuum. Next, gerstleyite flakes are mechanically exfoliated using Nitto tape (SPV 224) from naturally occurring bulk gerstleyite mineral (from Western Mine, Boron, Kern County, California, USA). After completing the mechanical exfoliation several times using Nitto tape and Scotch tape, the gerstleyite thin flakes are transferred to the pretreated glass substrates followed by heat treatment at 115–120 °C for 5 min. Then, the exfoliated flakes are investigated using optical reflection and transmission microscope and atomic force microscope for estimating the surface smoothness, flake shape, size and thickness.

### Angle-resolved polarized Raman spectroscopy

The gerstleyite flake is illuminated with a 632.8 nm He–Ne laser using a 40× objective lens (NA = 0.65) and the back-reflected signal is collected using the same objective lens. The incident laser beam polarization is controlled by engaging a linear polarizer and a rotating half-wave plate in the illumination path. The collected signal is then routed to a spectrometer (Horiba, iHR 520) using a beam splitter and a set of mirrors. The back-scattered light is also passed through the corresponding edge filter (Semrock, LP02-633RE-25) in the collection path for rejecting the excitation laser light. Afterwards, the signal is passed through a linear polarization analyzer in the collection path to record the parallel polarization components of the Raman spectra.

### Polarization-resolved optical absorption spectroscopy

A broadband white light source (Thorlabs, SLS201L, 360–2600 nm) is passed through a linear polarizer and a half-wave plate and then focused on the probed gerstleyite flake with a 80× objective lens (NA = 0.5). To record the reflection spectrum, the back‐reflected light is collected from the probed flake using the same objective lens and routed towards the spectrometer using a beam splitter and a set of mirrors. Similarly, the source spectrum is measured by mounting a silver mirror on the sample stage to perform the normalization process for obtaining the reflectance (*R*) spectrum. For the transmission spectrum, the transmitted light through the flake is collected from the other side of glass substrate using another 100× objective lens (NA = 0.7) and routed towards the spectrometer with mirrors. In the transmission configuration, the source spectrum is recorded by taking out the sample from the excitation path. The transmittance (*T*) spectrum is then obtained by normalizing the sample response by the source spectrum. Finally, the absorbance (*A*) spectrum is achieved using the relation of *A* = 1 − *R* − *T*.

### Birefringence measurement

A broadband white light source is passed through a linear polarizer (400–800 nm) and focused on the investigated gerstleyite flake with a 20× objective lens (NA = 0.42). The transmitted light is collected from the other side of glass substrate using another 50× objective lens (NA = 0.42) and routed to a charge-coupled device (CCD) camera to record the image. The linear polarization of incident white light is rotated from 0° to 180° with a half-wave plate, while a linear polarization analyzer is introduced in the collection path for maintaining the cross-polarization illumination configuration for each incident linear polarization angle. Other experimental conditions such as pinhole size and camera exposure time is kept identical during the measurement. The captured images are further processed for removing the background contribution and extracting the brightness contrast between the gerstleyite crystal and the glass substrate. Lastly, the normalized light intensity from the flake is plotted depending on the incident linear polarization angle to probe the birefringence effect.

### Third-harmonic generation measurement

The sample is excited by a pulsed laser beam at the wavelength of 1560 nm from a femtosecond laser source (pulse width 90 fs, repetition rate 80 MHz) using a 40× objective lens (NA = 0.65). The THG emission is collected in the transmission configuration from the other side of glass substrate using a 100× objective lens (NA = 0.7). A shortpass filter is used in the collection path for rejecting the laser beam. The filtered signal is then routed towards a spectrometer (Horiba, iHR 520) and a CCD camera for recording the spectra and corresponding image.
